# The ratio of CRP to prealbumin levels predict mortality in patients with hospital-acquired acute kidney injury

**DOI:** 10.1186/1471-2369-12-30

**Published:** 2011-06-29

**Authors:** Qionghong Xie, Ying Zhou, Zhongye Xu, Yanjiao Yang, Dingwei Kuang, Huaizhou You, Shuai Ma, Chuanming Hao, Yong Gu, Shanyan Lin, Feng Ding

**Affiliations:** 1 1Division of Nephrology, Huashan Hospital, Fudan University, Shanghai 200040, China

**Keywords:** inflammation, malnutrition, CRP, prealbumin, acute kidney injury

## Abstract

**Background:**

Animal and human studies suggest that inflammation and malnutrition are common in acute kidney injury (AKI) patients. However, only a few studies reported CRP, a marker of inflammation, albumin, prealbumin and cholesterol, markers of nutritional status were associated with the prognosis of AKI patients. No study examined whether the combination of inflammatory and nutritional markers could predict the mortality of AKI patients.

**Methods:**

155 patients with hospital-acquired AKI were recruited to this prospective cohort study according to RIFLE (Risk, Injury, Failure, Lost or End Stage Kidney) criteria. C-reactive protein (CRP), and the nutritional markers (albumin, prealbumin and cholesterol) measured at nephrology consultation were analyzed in relation to all cause mortality of these patients. In addition, CRP and prealbumin were also measured in healthy controls (n = 45), maintenance hemodialysis (n = 70) and peritoneal dialysis patients (n = 50) and then compared with AKI patients.

**Results:**

Compared with healthy controls and end-stage renal disease patients on maintenance hemodialysis or peritoneal dialysis, patients with AKI had significantly higher levels of CRP/prealbumin (*p *< 0.001). Higher level of serum CRP and lower levels of albumin, prealbumin and cholesterol were found to be significant in the patients with AKI who died within 28 days than those who survived >28 days. Similarly, the combined factors including the ratio of CRP to albumin (CRP/albumin), CRP/prealbumin and CRP/cholesterol were also significantly higher in the former group (*p *< 0.001 for all). Multivariate analysis (Cox regression) revealed that CRP/prealbumin was independently associated with mortality after adjustment for age, gender, sepsis and sequential organ failure assessment (SOFA, *p *= 0.027) while the others (CRP, albumin, prealbumin, cholesterol, CRP/albumin and CRP/cholesterol) became non-significantly associated. The hazard ratio was 1.00 (reference), 1.85, 2.25 and 3.89 for CRP/prealbumin increasing according to quartiles (*p *= 0.01 for the trend).

**Conclusions:**

Inflammation and malnutrition were common in patients with AKI. Higher level of the ratio of CRP to prealbumin was associated with mortality of AKI patients independent of the severity of illness and it may be a valuable addition to SOFA score to independent of the severity of illness and it may be a valuable addition to SOFA score to predict the prognosis of AKI patients.

## Background

Acute kidney injury (AKI) is well-known associated with increased mortality of in hospital patients. Although great advances in diagnosis of AKI have been made, the mortality remains high [1-5]. Among the patients with AKI, inflammation and malnutrition were common [6,7], but there were only a few studies suggested that inflammation and malnutrition were associated with the prognosis of AKI patients [7,8].

Serum C reactive protein (CRP) was an acute-phase protein synthesized by the liver following stimulus by various cytokines, markedly increased within hours after infection or inflammation. The relatively short half-life of approximately 19 hours makes it a useful monitor for infection and inflammatory disease. In addition, laboratory tests for CRP are easily available and less costly than cytokine tests [9-11]. Some studies suggested that increased CRP level was associated with sepsis and mortality of critical illness [9]. However, no study demonstrated that CRP was a predictor for mortality of AKI patients [12]. Malnutrition was another outstanding problem in AKI patients and has been paid attention to in recent years [13-16]. According to the International Society of Renal Nutrition and Metabolism (ISRNM), the serum chemistry markers including albumin, prealbumin and cholesterol are recommended to assess the nutritional status [17]. Chertow et al reported that prealbumin is as important as albumin in the nutritional assessment of hemodialysis patients [18]. The study by Cano et al showed that an improvement in prealbumin during nutritional therapy was associated with a decrease in morbidity and mortality in malnourished hemodialysis patients [19]. Another study also reported that even though baseline serum prealbumin may not be superior to albumin in predicting mortality in maintenance hemodialysis (MHD) patients, prealbumin concentrations <20 mg/dL were associated with death risk in those patients and a fall in serum prealbumin over 6 month was independently associated with increased death risk [20]. In AKI patients, Perez-Valdivieso et al found that serum prealbumin levels <11 mg/dL were strongly associated with a higher risk of death independent of AKI severity [21]. Besides, few studies reported the predict value of prealbumin in AKI patients. Cholesterol is another nutritional biomarker wildly used in clinic. In 1994, Dunham et al found that patients with severe trauma had a sudden reduction in total serum cholesterol concentration [22]. Hypocholesterolemia has been observed in patients undergoing surgical interventions and in those with multiple-organ dysfunction syndrome [23,24]. However, few studies showed the association between cholesterol and the prognosis of AKI patients. In addition, the correlation between inflammation and malnutrition was close and complex because inflammation could lead to malnutrition, as well as malnutrition was an adverse factor for the control of inflammation [15]. Pinilla et al reported that the ratio of CRP to prealbumin (CRP/prealbumin) was associated with the severity of organ dysfunction in critically ill patients [25]. However, no study reported that the combination of the inflammatory and nutritional markers could predict the mortality of AKI patients. The aim of our study was to investigate the correlation between inflammatory marker (CRP), nutritional markers (albumin, prealbumin and cholesterol) and 90 days mortality of AKI patients. We also studied the combination of inflammatory and nutritional markers including the ratio of CRP to albumin (CRP/albumin), CRP/prealbumin and CRP/cholesterol as tools to assess the risk for death in patients with AKI.

## Methods

### Subjects

We prospectively studied a random cohort of adult patients with hospital-acquired acute kidney injury (AKI) from December 2008 to November 2009 at Huashan hospital affiliated to Fudan university, a tertiary hospital including 30 wards, comprising 1500 beds in the city of Shanghai, China. AKI was determined using the RIFLE (Risk, Injury, Failure, Loss, or End stage kidney) staging criteria for changes in the serum creatinine within one week [26]. In brief, a RIFLE risk was defined by a 50% rise in creatinine, RIFLE injury by a doubling of baseline creatinine, and RIFLE failure by 3 folds increase of baseline creatinine. In-patient's serum creatinine data were monitored in our hospital's information system daily. Patients with creatinine levels rising within one week in accordance with RIFLE criteria were consulted by nephrologists within 24 hours. Those with AKI caused by post-renal obstruction, glomerulonephritis, interstitial nephritis or vasculitis, etc were excluded from the study. Patients were followed prospectively from the time of nephrology consultation until death. The observational period was 3 months and the primary outcome was all-cause mortality. We also compared results obtained in AKI patients with healthy controls (n = 45) and end-stage renal disease (ESRD) patients on maintenance hemodialysis (n = 55) or continuous ambulatory peritoneal dialysis (n = 50). The healthy control was randomly selected in healthcare center of Huashan Hospital, and the stable MHD patients and PD patients were randomly selected from hemodialysis center or peritoneal dialysis center of Huashan Hospital. The study was approved by the ethics committee of Huashan Hospital, Fudan University (approval number: 2009-097). All patients gave written informed consent.

### Clinical Data

In the first 24 hours after AKI was diagnosed, the variables including demographic data, presence of sepsis, need for mechanical ventilation, AKI pathogenesis (ischemic, nephrotoxic or multifaceted), comorbidity conditions, primary and concomitant diagnoses were recorded.

The severity of illness was evaluated according to the sequential organ failure assessment (SOFA) score [27]. The SOFA score included six systems which were respiratory, cardiovascular, neurologic, renal, hematologic and hepatic system. Organ failure was defined as a score of ≥3 for each. Sepsis was defined when the patient met their criteria for systemic inflammatory response syndrome (SIRS) and simultaneously presented with a documented or a suspected infection. Infection was diagnosed according to usual clinical, laboratory and microbiological parameters. The criteria for SIRS derived from 1992 the American College of Chest Physicians/Society of Critical Care Medicine Consensus Conference [28].

### Blood samples

The samples for measurement were fasting blood and obtained within 24 hours after AKI diagnosis, pre-dialysis for maintenance hemodialysis patients and random for peritoneal patients and healthy controls. The blood was collected in serum-separating tubes and the serum was separated within 30 min of sample collection, and then aliquoted and stored at -80°. CRP were measured by nephelometry immunoassay, the detectable limit of CRP was 0.316 mg/dL. The levels of CRP below the detectable limit were assumed as 0.158 mg/dL. Serum albumin, prealbumin and cholesterol were measured using the automatic biochemistry analyzer.

### Statistical analysis

Continuous variables were expressed as mean ± SD or median with range of quartile, and categorical variables were expressed as percentage. In univariate analysis, comparisons between groups were analyzed using independent t test or Mann-Whitney U test for continuous variables, and Pearson chi test or Fisher exact test for categorical variables. In multivariate analysis, Cox proportional hazards regression was used to identify independent predictors of mortality in AKI patients. Covariates including age, gender, sepsis and SOFA were used for stepwise adjustment. The Hazard ratio (HR) for death was expressed per 2 mg/dL increase in CRP, 5 mg/dL decrease in prealbumin, 0.5 g/dL decrease in albumin, 3 mg/dL decrease in cholesterol, 0.1 increase in CRP (mg/dL)/prealbumin (mg/dL), 1 increase in CRP (mg/dL)/albumin (g/dL), 0.1 increase in CRP (mg/dL)/cholesterol (mg/dL).

The Kaplan-Meier survival curve at 90 days was used to evaluate the difference between the 2 groups which were divided according to the median of CRP/prealbumin value, and compared using log-rank test. The time of origin was the day when nephrology consultation began. The event was defined death and the cases lost to follow-up were censored at their last observation. All statistical tests were 2-sided, and differences were considered statistically significant with *p *value < 0.05.

## Results

### Patient characteristics

A total of 155 AKI patients with mean age (±SD) 63.4 (±18.4) years were enrolled into this study, and 74.2% of which were male. The predominant comorbidities were as follows: hypertension in 66 patients (44.60%), diabetes in 25 patients (16.9%) and CKD in 10 patients (6.8%). The basic serum creatinine was 0.9 (0.7-1.1) mg/dL and raised to 1.9 (1.5-2.7) mg/dL when AKI was diagnosed according to RIFLE staging with risk 71 patients (45.8%), injury 38 patients (24.5%) and failure 46 patients (29.7%). The etiologies of AKI were ischemia (42.6%), nephrotoxicity (20.0%), and multifaceted factors (37.4%). Of the 155 patients with AKI, 67 patients (43.8%) suffered from sepsis, 31 patients (20.0%) needed ventilation and 30 patients (19.4%) needed dialysis during the following period. The SOFA score which indicated the severity of illness was 7 (4-11). The overall 28 days mortality was 33.5%.

Table 1 also displayed the demographic and clinical features of survivors (n = 103) and non-survivors (n = 52) which were grouped according to the survival status at 28 days. There were no significant differences in age, gender, basic serum creatinine, RIFLE criteria and etiology of AKI between the the two groups. The comparison showed that survival group had higher rate of CKD history but lower serum creatinine level when AKI diagnosed. Unsurprising, the median of SOFA score as assessed at the time of nephrology consultation were significantly higher in the non-survival group (*p *< 0.001). During the course of the study, the need for ventilation was less among survivors (11.7% vs. 36.5%, *p *< 0.001) while the need for renal replacement therapy was similar in the two groups (17.5% of survivors vs. 23.1% of nonsurvivors, *p *= 0.405). No differences were also found in white blood cell count, bilirubin and hemoglobin.

**Table 1:** Demographic and clinical data of patients at the time of acute kidney injury diagnosis

	Total (n = 155)	Survivors (n = 103)	Non-survivors (n = 52)	*p *value
Age	63.4 ± 18.4	63.96 ± 19.04	62.2 ± 17.1	0.577
Gender (male)	115(74.2%)	78 (75.7%)	37 (71.2%)	0.539
Basic Scr(μmol/L)	79.5 (61.0-100.0)	78.5 (61.3-98.5)	80.5 (58.8-102.0)	0.501
Scr when AKI diagnosed (μmol/L)	165 (132-237)	158 (126-216)	190.5 (142.5-241.5)	0.030
RIFLE criteria (%)				0.235
Risk	71 (45.8%)	50 (48.5%)	21 (40.4%)	
Injury	38 (24.5%)	27 (26.2%)	11 (21.2%)	
Failure	46 (29.7%)	26 (25.2%)	20 (38.5%)	
Etiology of AKI				0.513
Ischemic	66 (42.6%)	44 (42.7%)	22 (42.3%)	
Multifaceted	58 (37.4%)	36 (35.0%)	22 (42.3%)	
Nephrotoxic	31 (20.0%)	23 (22.3%)	8 (15.4%)	
Comorbidity				
Hypertension	66 (44.6%)	40 (40.8%)	26 (52.0%)	0.195
Diabetes	25 (16.9%)	19 (19.4%)	6 (12.0%)	0.257
CKD	10 (6.8%)	10 (10.2%)	0 (0%)	0.017
Sepsis	67 (43.8%)	41 (40.2%)	26 (51.0%)	0.205
Ventilation	31 (20.0%)	12 (11.7%)	19 (36.5%)	<0.001
Dialysis	30 (19.4%)	18 (17.5%)	12 (23.1%)	0.405
Hemoglobin (g/dL)	11.2 (9.6-12.6)	11.2 ± 2.0	10.7 ± 3.1	0.265
WBC (10^3^/mm^3^)	11.2 (7.7-16.5)	10.8 (7.1-16.9)	12.1 (9.2-16.4)	0.222
TB (mmol/L)	12.0 (8.2-21.2)	12.0 (8.0-18.5)	13.0 (8.5-28.4)	0.112
SOFA	7 (4-11)	4 (3-7)	12.5 (9.3-15.0)	<0.001

Data were divided into two groups according to survival status of 28 days.

Abbreviations: Scr: serum creatinine; AKI, acute kidney injury; CKD, chronic kidney disease; WBC, white blood cell; TB: Total bililubin; SOFA, sequential organ failure assessment.

### The inflammatory and nutritional markers and their relationship with mortality of AKI patients

CRP, albumin, prealbumin and cholesterol determined from blood obtained within 24 hours of AKI diagnosis were displayed in table 2. CRP, reflecting the inflammation status, was elevated in non-survivors compared with the survivors (6.6 (1.9 to 11.9) vs. 3.8 (1.2 to 11.4) mg/dL, *p *= 0.001). On the contrary, albumin, prealbumin and cholesterol which reflecting the nutritional status were all significantly lower in non-survivors (3.1 ± 0.6 vs. 3.3 ± 0.7 mg/dL, *p *= 0.025 for albumin; 12.5 ± 5.9 vs. 16.4 ± 6.9 mg/dL, *p *= 0.001 for prealbumin; 12.2 ± 5.0 vs. 14.5 ± 6.1 mg/dL, *p *= 0.020 for cholesterol). Further analysis showed that the combined factors including CRP/albumin, CRP/prealbumin and CRP/cholesterol were all significantly higher in non-survivors when compared with the survivors (*p *< 0.001).

**Table 2:** Univariate analysis of selected possible predictors of mortality in patients with AKI

	Total	survivors	nonsurvivors	*p *value
Albumin (g/dL)	3.2 ± 0.7	3.3 ± 0.7	3.1 ± 0.6	0.025
Prealbumin (mg/dL)	15.1 ± 6.8	16.4 ± 6.9	12.5 ± 5.9	0.001
Cholesterol (mg/dL)	13.9 ± 5.8	14.5 ± 6.1	12.2 ± 5.0	0.020
CRP (mg/dL)	6.6 (1.9-11.9)	3.8 (1.2-11.4)	10.6 (3.1-15.1)	0.001
CRP: Albumin (mg/dL: g/dL)	1.97 (0.56-4.17)	1.36 (0.35-3.57)	3.57 (0.94-5.18)	0.001
CRP: Prealbumin (mg/dL: mg/dL)	0.42 (0.11-1.13)	0.34 (0.07-0.84)	1.04 (0.22-1.55)	<0.001
CRP: Cholesterol (mg/dL: mg/dL)	0.53 (0.13-1.06)	0.38 (0.07-0.80)	0.87 (0.30-1.46)	<0.001

Abbreviation: CRP, C reactive protein.

**Figure 1:**
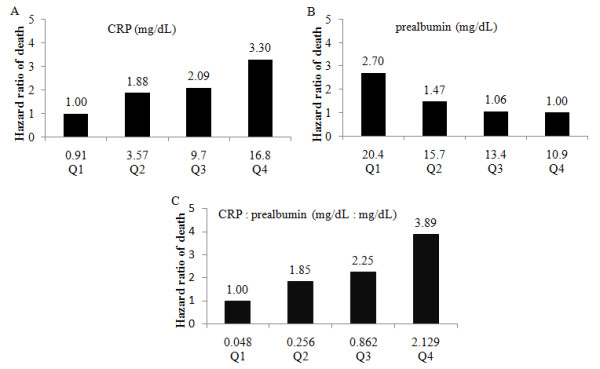
**(A) Risk profiles for CRP according to quartiles, p = 0.029 for the trend; (B) Risk profiles for prealbumin, p = 0.013 for the trend; (C) Risk profile for CRP: prealbumin, p = 0.01 for the trend**. Values along horizontal axis represent the means of CRP, prealbumin and CRP/prealbumin of the respective quartiles. Hazard ratios of death for CRP rising, prealbumin decreasing and CRP/prealbumin rising according to quartiles were adjusted by age and gender.

**Figure 2:**
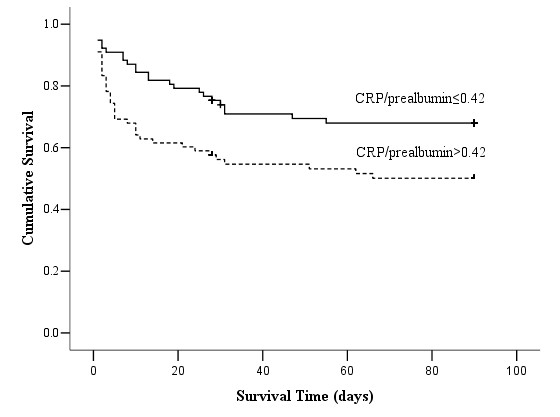
**Kaplan-Meier analysis for the cumulative percentage of surviving patients at 90 days according to different CRP/prealbumin levels**. The median of CRP/prealbumin was used as the cut-off point to divide the patients with AKI into 2 groups. Patients with higher levels of CRP/prealbumin (n = 78) had a significantly lower survival rate in these patients (log rank test, *p *< 0.01).

In multivariable analysis of these selected possible predictors for mortality of AKI patients, we controlled the demographic factors including age and gender as model 1, controlled age, gender and sepsis as model 2, controlled age, gender and SOFA as model 3, controlled age, gender, sepsis and SOFA as model 4. Table 3 showed that when adjusted for the severity of illness (SOFA), CRP/prealbumin remained an independent predictor of mortality (*p *= 0.027) while the others were no longer independently associated. Hazard ratio was 1.037 per 0.1 increases for CRP/prealbumin when adjusted for age, gender, sepsis and SOFA. Figure 1 illustrates the risk profiles for CRP/prealbumin according to quartiles. The values for CRP/prealbumin above the 25^th ^percentile were associated with a progressively higher risk of mortality after adjusting for age and gender (*p *= 0.01 for the trend). We also used the median of CRP/prealbumin as cut-off point to divide the patients into 2 groups and made the Kaplan-Meier plot (Figure 2) which showed that the survival of patients with CRP/prealbumin >0.42 was significantly worse than that of patients with lower levels (log rank test, *p *< 0.01). At the end of the follow-up period, the mortality was remarkably higher in the CRP/prealbumin >0.42 group (63.4% vs. 38.7%, *p *= 0.026).

**Table 3:** Multivariate Cox regression analysis for the selected possible predictors of mortality in patients with AKI

	CRP (mg/L)		prealbumin (mg/dL)		CRP/prealbumin		albumin (g/dL)		CRP/albumin		cholesterol (mg/dL)		CRP/cholesterol	
	
	HR	*p*	HR	*p*	HR	*p*	HR	*p*	HR	*p*	HR	*p*	HR	*p*
unadjusted	1.108	0.006	1.404	0.002	1.057	0.000	1.185	0.075	1.128	0.022	1.154	0.043	1.039	0.001
model 1	1.123	0.003	1.398	0.002	1.059	0.000	1.185	0.083	1.137	0.017	1.166	0.041	1.043	0.001
model 2	1.121	0.005	1.404	0.002	1.058	0.000	1.166	0.121	1.132	0.027	1.171	0.034	1.043	0.001
model 3	1.052	0.285	1.100	0.407	1.037	0.023	0.974	0.785	1.029	0.653	0.947	0.453	0.993	0.633
model 4	1.051	0.320	1.099	0.414	1.037	0.027	0.967	0.737	1.027	0.681	0.949	0.470	0.991	0.588

The Hazard ratio (HR) for death was expressed per a 2 mg/dL increase in CRP, 5 mg/dL decrease in prealbumin, 0.5 g/dL decrease in albumin, 3 mg/dL decrease in cholesterol, 0.1 increase in CRP (mg/dL)/prealbumin (mg/dL), 1 increase in CRP (mg/dL)/albumin (g/dL), 0.1 increase in CRP (mg/dL)/cholesterol (mg/dL) for a univariate model and sequential adjustment models.

The multivariate analysis sequentially adjusted models for covariates as follows: Model 1, adjusted for age, gender; Model 2, adjusted for age, gender and sepsis; Model 3, adjusted for age, gender and SOFA; Model 4, adjusted for age, gender, sepsis and SOFA.

### The ratio of CRP to prealbumin dramatically increased in patients with AKI

**Figure 3:**
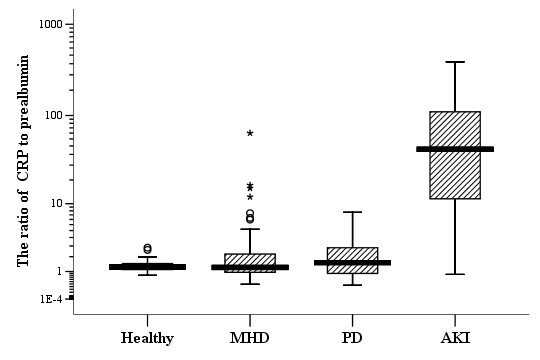
**Levels of the ratio of CRP to prealbumin in different populations**. Healthy (n = 45); MHD: maintenance hemodialysis (n = 70); PD: peritoneal dialysis (n = 50); AKI: acute kidney injury (n = 155). The units of CRP and prealbumin were both mg/dL. Mann-Whitney U test was used for comparison between the populations, *p *< 0.001. Horizontal bars indicate 10th, 25th, 50th (median), 75th, and 90th percentile levels.

Patients with AKI had marked increase in serum CRP levels and decrease in prealbumin simultaneously when compared with healthy control subjects (6.56 vs. 0.16 mg/dL for median of CRP, *p *< 0.001; 15.1 ± 6.8 vs. 25.8 ± 5.5 mg/dL for prealbumin, *p *< 0.001). Similarly, the value of CRP was 10- to 20- fold higher in the AKI patients compared with ESRD patients on maintenance hemodialysis or peritoneal dialysis. The prealbumin levels in the AKI patients were also significantly lower than ESRD patients (29.9 ± 5.9 mg/dL for MHD patients; 31.1 ± 8.5 mg/dL for PD patients). As illustrated in figure 3, the CRP/prealbumin was also dramatically increased in AKI patients (*p *< 0.001).

## Discussion

The aim of this study was to assess whether serum inflammatory and nutritional markers can predict mortality in a cohort of patients with AKI. The inflammatory marker CRP, the nutritional markers including albumin, prealbumin, cholesterol and their ratio including CRP/albumin, CRP/prealbumin, CRP/cholesterol were analyzed. The results showed that after adjusting for gender and age, all of the markers were associated with the 90 days mortality in these patients. However, when further adjustment was done for the severity of illness, only CRP/prealbumin remained significant. We then used the median of CRP/prealbumin in this cohort as the cut-off point to separate patients into two groups which showed that higher CRP/prealbumin had a higher mortality of 90 days. Such results suggested that when AKI was diagnosed, higher levels of CRP/prealbumin in patients with AKI indicated, at least partly, a poorer prognosis and more aggressive diagnostic and therapeutic interventions were needed to avoid complications.

Consistent with previous studies, malnutrition and inflammation was common in our patients with AKI [7,29]. In Fiaccadori's study in 1999, severe malnutrition, defined by Subjective Global Assessment (SGA) class C, was documented in about 40% of patients [7]. According to the International Society of Renal Nutrition and Metabolism (ISRNM), the serum albumin <3.8 g/dL was recommended as a main diagnostic indicator of malnutrition [17]. Not surprisingly, approximate 80% patients in this cohort presented with serum albumin values <3.8 g/dL. Prealbumin is a nutritional marker used to evaluate recent nutritional status with a short half-life and a rapid synthesis rate [30-32], and it is also a negative acute-phase protein and inverse associated with inflammation. According to the Nutritional Care Consensus Group, serum prealbumin >15 mg/dL indicates that patients are not at risk for malnutrition [32]. There were more than 50% patients with serum prealbumin <15 mg/dL in our study. CRP is one of the wildly used biomarkers for monitoring the course of infection and inflammation [9-11]. Compared with healthy control, MHD and PD patients, CRP in these patients with AKI were significantly higher.

Previous studies suggested that malnutrition and inflammation had a negative impact on the prognosis of AKI patients. The meta-analysis by Wiedermann et al. provided evidence that hypoalbuminemia is a significant independent predictor of death following AKI development [13]. Although the phenomenon that the lower albuminemia, the poorer prognosis was also found in our study, there was no statistical significance in univariate analysis (*p *= 0.075). This was probably because the sample size of our study was not large enough. Besides, there are only a few studies examined the association between CRP or prealbumin and mortality of these subjects [8,12,21] The PICARD study showed that there were no significant differences in CRP levels between acute renal failure (ARF) patients who died during hospitalization and survivals [8]. According to Valdivieso et al., serum prealbumin levels <11 mg/dL were strongly associated with a higher risk of death, independent of AKI severity, comorbidities and serum CRP [21]. Differently, all of the markers including serum albumin, prealbumin and cholesterol in the current study were not significantly associated with the 90 mortality after adjustment for the severity of illness.

Pinilla et al demonstrated a strong correlation between the ratio of CRP to prealbumin and the severity of organ dysfunction in critically ill patients [25]. However, the association of CRP/prealbumin and prognosis of AKI patients hasn't been reported. Our study suggested that CRP/prealbumin when AKI was diagnosed was independently associated with mortality of these patients after adjustment for the severity of illness. CRP/prealbumin levels >3 having a particularly higher mortality suggested that inflammation activity and malnutrition indicated a poor prognosis. These data provide evidence supporting the measurement of serum CRP/prealbumin levels may be an inexpensive and useful tool in the evaluation of the risk profiles of AKI patients.

In addition, 43.8% of the AKI patients suffered from sepsis in our study. However, septic AKI obviously did not play a role in AKI prognosis. This is different from many previous studies. The PICARD study showed that sepsis in AKI patients portended a poor prognosis, with higher mortality rates and relatively longer length of stay [33]. Different study population may contribute to the discrepancy that our patients were not confined to the ICU or critical ill patients. There were some limitations in this study. Firstly, this was an observational, single-center and relatively small size study, and our results only described the association between inflammatory or nutritional markers and 90 days mortality. Secondly, the studied population was composed by heterogeneous AKI patients in a tertiary comprehensive hospital. Selection bias may have influence the result. At the same time, urinary output data were not available, so it was not possible to estimate the RIFLE criteria according to the values.

## Conclusions

In conclusion, the present study firstly assesses the correlation between CRP/prealbumin levels and the mortality in AKI patients. Lower serum CRP/prealbumin levels were strongly associated with higher mortality after adjusting for the severity of illness (SOFA). So it was a good marker of mortality in these patients. In addition, the measurement of CRP and prealbumin were inexpensive and it may be a valuable addition to SOFA scores to predict the risk of death in AKI patients.

## Competing interests

The authors declare that they have no competing interests.

## Authors' contributions

XQ collected data, analyzed, interpreted data and drafted the manuscript. ZY collected data and drafted the manuscript. XZ, YY, KD, YH and MS collected data and analyzed. HC, GY and LS collected data and coordinated and revised the manuscript. DF conceived the study, participated in its design and coordination and helped to draft the manuscript and had full access to all the study data and assume responsibility for the integrity of the data and the accuracy of the analysis. All authors read and approved the final manuscript.

## Pre-publication history

The pre-publication history for this paper can be accessed here:

http://www.biomedcentral.com/1471-2369/12/30/prepub
